# The Impact of Tropospheric Anomalies on Sea-Based JPALS Integrity

**DOI:** 10.3390/s18082579

**Published:** 2018-08-07

**Authors:** Yue Zhang, Zhipeng Wang

**Affiliations:** School of Electronic Information Engineering, Beihang University, Beijing 100191, China; zhangyue9405@buaa.edu.cn

**Keywords:** Sea-Based JPALS, troposphere delay, tropospheric duct anomaly, non-nominal troposphere, VPL

## Abstract

The Joint Precision Approach Landing System (JPALS) addresses tropospheric errors through double-difference and tropospheric model correction. Large residuals occur with two types of tropospheric anomalies: the vertical duct and horizontal non-nominal troposphere. Through analyzing 8 years of meteorological data from the European Center for Medium-Range Weather Forecasts (ECMWF), we find that the two types of anomalies can occur simultaneously. In addition, the existing vertical protection level (VPL) calculation method under tropospheric anomalies is based on the least squares method, which is not applicable to Sea-Based JPALS using the Kalman filter. Therefore, we start by calculating the zenith duct error by numerical integration. The maximum error observed is 45.64 mm, and the error seasonal characteristic is analyzed. For the non-nominal troposphere, the worst meteorological conditions in the Chinese surrounding sea areas are used to calculate the non-nominal errors, which are fitted to a satellite-elevation-dependent model. Then, a VPL calculation method based on the Kalman filter under tropospheric anomalies is proposed. Finally, a multiple approach simulation is conducted. The results show that the average VPL increments introduced by the duct and non-nominal troposphere anomalies are 0.082 m and 0.211 m, respectively, with growth percentages of 12.903% and 30.857%, respectively. The increment under simultaneous anomalies is 0.272 m with a growth of 40.427%. Furthermore, the average availability under normal conditions is 100%. Considering the duct and the non-nominal troposphere anomalies, the availability loss is 0.017% and 3.674%, respectively. Under simultaneous anomalies, this loss is 4.743%.

## 1. Introduction

The troposphere is the lower part of the atmosphere over the earth’s surface, extending from the surface to a height of approximately 16 km. Tropospheric refraction causes delays in satellite signal transmission. The amount of tropospheric delay in the zenith direction is approximately 2.3 m, which results in a slant delay of approximately 23.5 m for a satellite with an elevation of 5° [1].

Unlike the ionosphere, the troposphere is a non-dispersive medium at Global Navigation Satellite System (GNSS) carrier frequencies. That is, the tropospheric impacts on GNSS signal transmission are independent of the working frequency and thus cannot be eliminated using dual or multiple frequency technology [2]. Therefore, the tropospheric delay is an important error source in precise GNSS applications.

Under normal conditions, the majority of the tropospheric delay can be removed by a tropospheric model. Several models exist that describe the nominal tropospheric delay under nominal conditions, including the Hopfield, Modified Hopfield, and Saastamoinen models [2,3]. In differential augmentation systems, such as the Ground Based Augmentation System (GBAS) and Joint Precision Approach Landing System (JPALS), under normal conditions, the meteorological conditions at the airport and ship are similar, and the tropospheric delay can be nearly eliminated by differential processing. However, under severe weather conditions such as heavy rainfall, tropospheric anomalies may occur, and the tropospheric errors cannot be completely eliminated by this method, resulting in differential residual tropospheric errors. The residual errors adversely affect the system accuracy and integrity performance [4]. Recent observations showing unexpected atmospheric behavior were reported at the 2014 International Civil Aviation Organization Navigation System Panel meeting and subsequently confirmed by the Federal Aviation Administration (FAA) and Boeing. The observations showed that significant spatial gradients with no link to ionosphere activity could be related to a non-modelled behavior of the troposphere [5,6].

Most of the previous studies in this context were conducted on GBAS, and there are few results on the impacts of tropospheric anomalies on the integrity of Sea-Based JPALS. JPALS is a GNSS-based military all-weather high-accuracy precision approach and landing system developed by the United States; it encompasses Land-Based JPALS and Sea-Based JPALS [7,8]. For aircraft landing, Sea-Based JPALS must provide higher accuracy and integrity performance than land-based applications due to the mobility of the reference station. Due to the highly stringent requirements, Sea-Based JPALS is generally based on carrier-phase differential positioning and Kalman filtering [9], so the corresponding protection level calculation method under the tropospheric anomaly hypothesis is different from that of GBAS.

**Figure 1:**
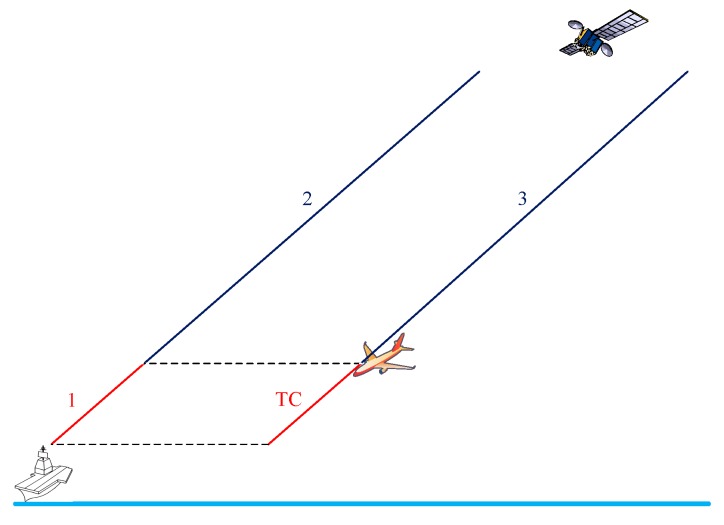
Tropospheric delay paths.

As shown in Figure 1, three tropospheric delay components are critical to Sea-Based JPALS. The total tropospheric delay observed at the ship station is composed of the delay over paths 1 and 2 (defined by the aircraft height). However, the aircraft, being at a higher height, is subject only to a delay over path 3. Under nominal conditions, the delays over paths 2 and 3 are highly spatially correlated and can be almost eliminated by differential processing. For the delay over path 1, a simple empirically derived model is used for this tropospheric correction (TC) [10]. There are then two types of tropospheric anomalies that affect JPALS. First, the large difference between the actual tropospheric delay on path 1 and the TC model value causes the tropospheric duct anomaly error [11]. Second, the large horizontal troposphere gradient causes significantly different delays over paths 2 and 3, which is called the non-nominal troposphere.

In 2011, a three-parameter wedge model for estimating the duct anomaly error and three vertical protection level (VPL) calculation methods for considering the duct anomaly were proposed [11]. In 2015, Ecole National Aviation Civile proposed a protection level calculation method for the non-nominal tropospheric error for GBAS [10]. In 2017, a satellite selection method to reduce the impact of the non-nominal tropospheric error on the GBAS integrity was proposed [12].

The three-parameter wedge model assumes that once the duct ends, the refractivity index instantaneously recovers the nominal profile values, which may not be realistic. Moreover, the protection level calculation methods under tropospheric anomaly hypothesis proposed previously are based on the least squares method, which are not applicable to Sea-Based JPALS using Kalman filtering [13,14]. In addition, based on 8 years of data covering China and its surrounding areas obtained from the European Center for Medium-Range Weather Forecasts (ECMWF), we found that the two types of tropospheric anomalies can occur simultaneously. However, previous studies have not considered the impact of the simultaneous occurrence of both types of anomalies on JPALS.

Therefore, we first calculate the zenith tropospheric delay (ZTD) error caused by the duct anomaly based on the meteorological data by numerical integration. Second, for the non-nominal troposphere, the worst meteorological conditions in the surrounding sea areas of China are used to calculate the non-nominal errors. Third, a protection level calculation method based on Kalman filtering under tropospheric anomalies is proposed. Finally, a simulation of multiple landing approaches is conducted to analyze the impacts of individual and simultaneous occurrences of the duct and non-nominal troposphere anomalies on Sea-Based JPALS integrity.

## 2. Tropospheric Anomalies

The main causes of tropospheric duct anomaly are temperature inversion, evaporation ducts, air subsidence, and air advection. An evaporation duct typically occurs over large expanses of water, such as the Great Lakes. Air advection typically occurs over coastal regions [11]. For the non-nominal troposphere, the main causes are weather fronts and heavy rainfall. The two anomalies can occur at the same time.

### 2.1. Tropospheric Duct Anomaly

Under nominal tropospheric conditions, the pressure drops exponentially with height, and the temperature decreases with height at an approximate rate of 1 K/100 m (over the first few kilometers above sea level). As a result, the refractivity gradient over height is approximately −40/km [11]. The tropospheric delay on path 1 in Figure 1 can be accurately estimated using the TC model. However, under anomalous atmospheric conditions, tropospheric ducts can be generated that result in a large difference between the actual tropospheric delay on path 1 and the TC value, thus introducing residual TC errors.

**Figure 2:**
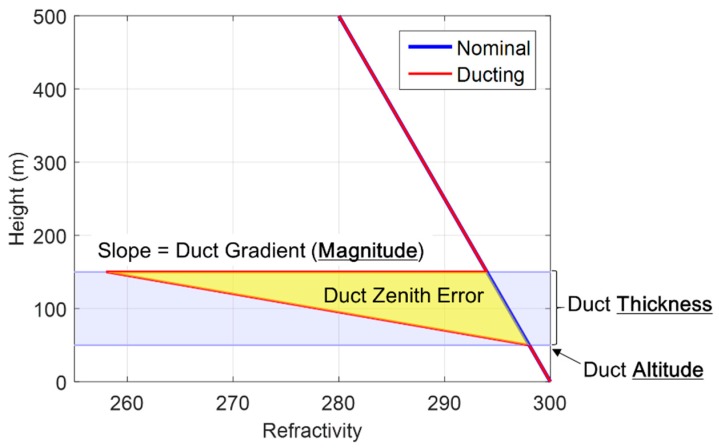
Three-parameter wedge model for duct anomaly [11].

Figure 2 shows the three-parameter wedge model for the duct anomaly proposed by the Illinois Institute of Technology. The blue and red lines are the refractive index under normal conditions and the duct anomaly, respectively. The area between the blue and red lines is the estimated zenith error caused by the duct anomaly. The model assumes that once the duct ends, the refractivity index instantaneously recovers the nominal profile values, which may not be realistic. Therefore, we just use the duct gradient to determine whether the duct anomaly exists. The error caused by the duct anomaly is calculated using a numerical integration method.

### 2.2. Non-Nominal Troposphere

**Figure 3:**
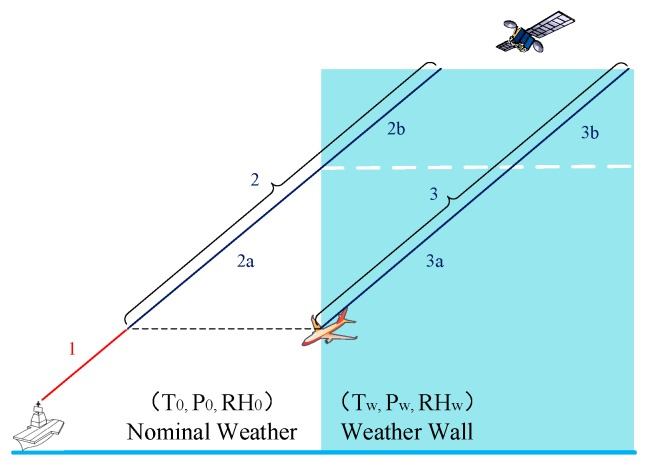
Weather Wall model to the right of the ship.

In 2008, Ohio University proposed a “Weather Wall model” for the non-nominal troposphere, as shown in Figure 3 [4]. In this model, the troposphere is partitioned by an infinite vertical plane, either side of which is defined as nominal ‘0’ and worst-case ‘w’ weather conditions for the temperature (T), pressure (P), and relative humidity (RH). In Figure 3, paths 2 and 3 are split into 2a, 2b and 3a, 3b, respectively. The split is made as the ship station signal leaves the worst-case weather wall. Path 3 is thus contained entirely within the weather wall with conditions (T_w_, P_w_, RH_w_), while path 2 to the ship experiences nominal conditions (T_0_, P_0_, RH_0_) during part 2a and during part 2b (T_w_, P_w_, RH_w_). The troposphere delay difference experienced by paths 2a and 3a is the non-nominal troposphere error. Without loss of generality, the weather wall can be on the right side of the aircraft or on the left side [6].

## 3. Tropospheric Error Estimation Based on ECMWF Data

Studies have shown that the ZTD derived from the ECMWF meteorological data by numerical integration has high precision [15]. Therefore, to study the above mentioned two types of tropospheric anomalies in China and the surrounding areas, the latest 8-year ECMWF ERA5 meteorological data from January 2010 to December 2017 are used.

The time resolution of the data is 1 h, i.e., the data correspond to 0, 1, 2, …, 23 UTC. The data have a horizontal resolution of 0.3° × 0.3° and a vertical resolution of 137 levels reaching 0.1 mbar at the top level. The latitude range of the data is from 3.5° N to 54° N, and the longitude range is from 73° E to 135.5° E.

In the aircraft landing application, tight integrity and accuracy requirements are only required close to the runway and at low altitudes (for example, a 500 m altitude) [16]. For Sea-Based JPALS, when the distance between the aircraft and ship decreases to 10 nm (the altitude of the aircraft is approximately 500 m), the aircraft enters the final approach area, where the aircraft would start receiving precision data for landing and precision relative navigation is needed. Also, stringent integrity and accuracy requirements are required [14,17]. If a duct exists at an altitude higher than the aircraft altitude (500 m), it will be eliminated in the differential process. Only ducts that are lower than the aircraft altitude will cause modeling errors that might jeopardize the integrity. Therefore, in this paper, only ducts occurring below 500 m are considered.

### 3.1. Tropospheric Duct Anomaly

**Figure 4:**
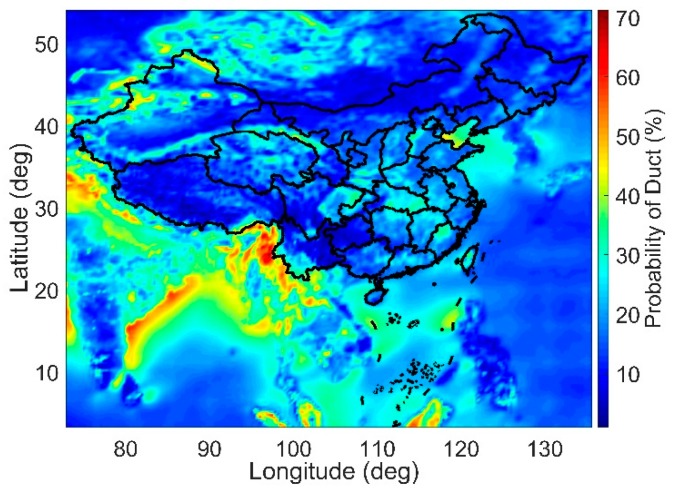
Map of the ducts’ likelihood of occurrence.

In this paper, the duct gradient is used to determine whether a duct occurs. That is, if the absolute value of the gradient of the refractivity index changes with altitude exceeds 100/km, then the duct anomaly is considered [11]. Figure 4 shows the map of the duct likelihood of occurrence using 8 years of ECMWF data (2010–2017). In this map, only ducts occurring below 500 m are considered. The map illustrates that the likelihood of a duct appearing below 500 m in some regions can reach 70%, and in the Bohai Sea it can reach approximately 45%. Therefore, to ensure the integrity of the system, the impact of troposphere ducts must be considered.

#### 3.1.1. Error Estimation Method

In the TC model, the refractivity *N*_model_ is expressed in terms of the reference refractivity *N_R_*, scale height *h*_0_ (unit: m) and antenna height *z* (unit: m) as follows:

(1) $$N_{\mathrm{model}}=N_{R}\exp\left(-z/h_{0}\right)$$

The model zenith tropospheric delay *ZTD*_model_ on path 1 is then evaluated by integrating *N*_model_ in Equation (1) from the height of the ship to that of the aircraft, which results in

(2) $$ZTD_{\mathrm{model}}=10^{-6}N_{R}h_{0}\left(1-e^{-\Delta h/h_{0}}\right)$$

where Δh is the height difference between the aircraft and ship (unit: m).

Then, the TC model is obtained by mapping the ZTD to the slant domain [18]:

(3) $$TC=N_{R}h_{0}\frac{10^{-6}}{\sqrt{0.002+\sin^{2}\left(\theta \right)}}\left(1-e^{-\Delta h/h_{0}}\right)$$

where *θ* is the satellite elevation (unit: rad).

The standard deviation $\sigma_{tropo}$ of *TC* is as follows:

(4) $$\sigma_{tropo}=\sigma_{N}\cdot TC/N_{R}$$

where $\sigma_{N}$ is the standard deviation of refractivity *N*.

The ship is assumed to be at the lowest level, and thus the temperature, pressure, and specific humidity (SH) of the lowest level are used to calculate *ZTD*_model_ in Equation (2).

Different from *N*_model_, the real refractivity index *N*_real_ is calculated using the temperature, pressure, and specific humidity [3]. The real zenith tropospheric delay *ZTD*_real_ on path 1 is calculated with ECMWF meteorological data using numerical integration.

(5) $$ZTD_{\mathrm{real}}=10^{-6}\sum_{i=0}^{M}N_{\mathrm{real},i}d_{i}$$

where $N_{\mathrm{real},i}$ is the real refractivity index at the *i*th level and calculated with the T*i*, P*i*, and SH*i* at that level. d is the height between levels. *M* is the number of levels below the aircraft altitude. The zenith duct error on path 1 is the difference between *ZTD*_real_ and *ZTD*_model_.

**Figure 5:**
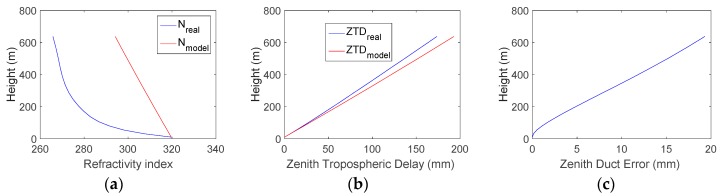
Example of a duct anomaly at location (37.8° N, 120.6° E) and time 12:00 on 1 June 2017. (**a**) shows *N*_real_ (blue curve) and *N*_model_ (red curve); (**b**) shows *ZTD*_real_ and *ZTD*_model_; and (**c**) shows the zenith duct error for this example.

Figure 5 shows an example of a duct anomaly. Figure 5a compares *N*_real_ (blue curve) and *N*_model_ (red curve) at a selected location (37.8° N, 120.6° E) in the presence of a duct at 12:00 on 1 June 2017. The refractivity gradient in the presence of a duct is much higher than the nominal case. Figure 5b shows *ZTD*_real_ and *ZTD*_model_, and Figure 5c shows the zenith duct error for this example. In Figure 5, the zenith duct error at 500 m is approximately 15 mm, which results in a slant error of approximately 11.49 cm for a satellite with an elevation of 7.5°. The duct error is much larger than the magnitude of the carrier-phase measurement noise. Therefore, the duct error is an important error source for Sea-Based JPALS.

#### 3.1.2. Results

**Figure 6:**
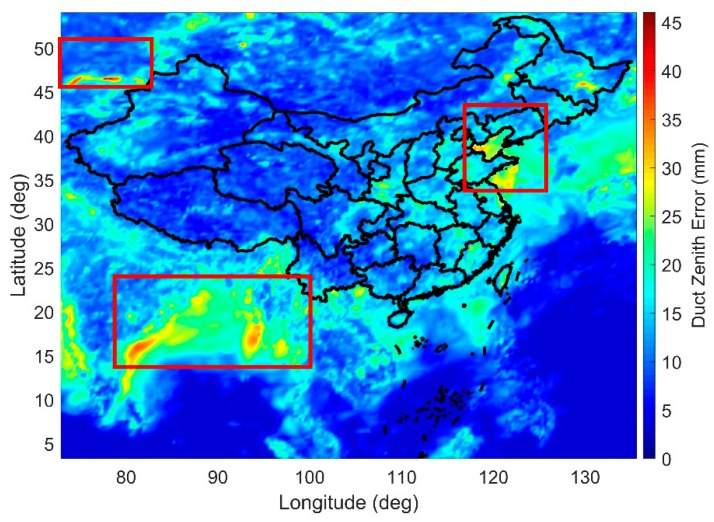
Map of the maximum zenith duct error at 500 m in China and the surrounding areas (2010–2017).

Figure 6 shows the map of the worst-case zenith duct error observed at each grid point in China and the surrounding areas during the period from 2010 to 2017. The large duct errors are mostly found at the junctions of land and water, such as the Balkhash Lake in Southeast Kazakhstan (upper left in Figure 6), the Bay of Bengal, the Andaman Bay (lower left in Figure 6), the Yellow Sea and the Bohai Sea (top right in Figure 6).

**Figure 7:**
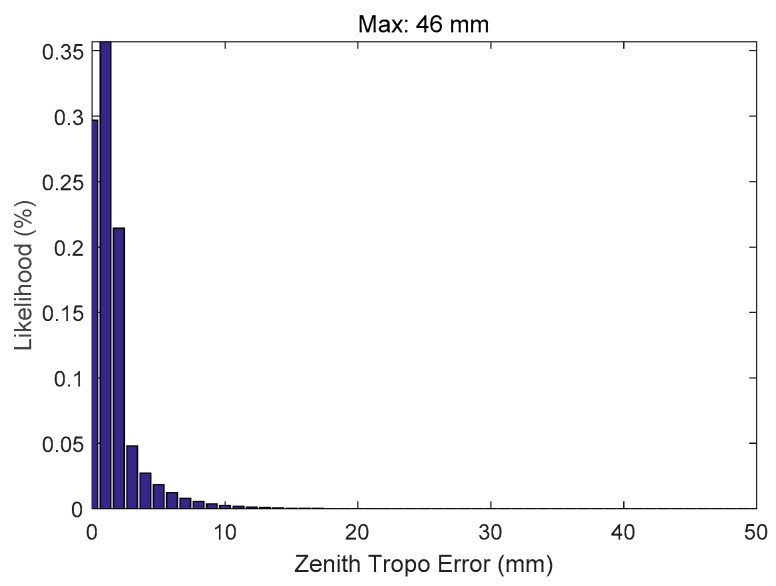
Histogram of the zenith duct error observed at 500 m.

To provide a better stochastic representation of the data, a histogram of the zenith duct error is shown in Figure 7. This histogram shows that the maximum error range that was observed at 500 m is 46 mm at the zenith (approximately 35.24 cm for a 7.5° elevation satellite).

The troposphere has prominent seasonal characteristics [15,19]. Therefore, we separately estimate the maximum duct errors in the four seasons of spring, summer, fall, and winter. The four seasons are divided by the spring equinox (21 March), summer solstice (22 June), fall equinox (23 September), and winter solstice (21 December).

**Figure 8:**
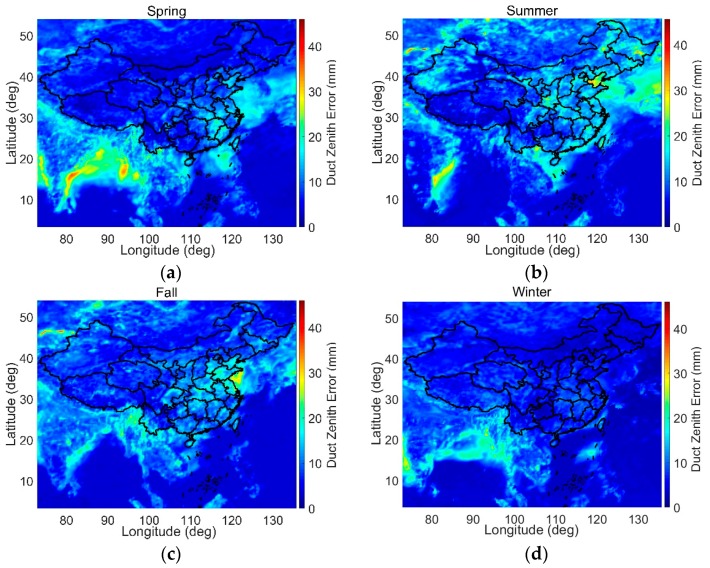
Maps of maximum zenith duct errors at 500 m for the four seasons. (**a**–**d**) show maps for the spring, summer, fall, and winter, respectively.

Figure 8 shows maps of the maximum zenith duct errors at 500 m for the four seasons. Different seasonal features appear for the duct anomaly in different places. In the Bay of Bengal and Andaman Bay, there are larger duct errors in the spring and winter than in the summer and fall. In Balkhash Lake, large duct errors occur in the summer and fall. In the Bohai Sea and Yellow Sea, large duct errors occur in the summer and fall, respectively. For the illustrated area, the maximum zenith duct errors in the four seasons of spring, summer, fall, and winter are 38.46 mm, 45.64 mm, 39.98 mm, and 32.98 mm, respectively.

**Figure 9:**
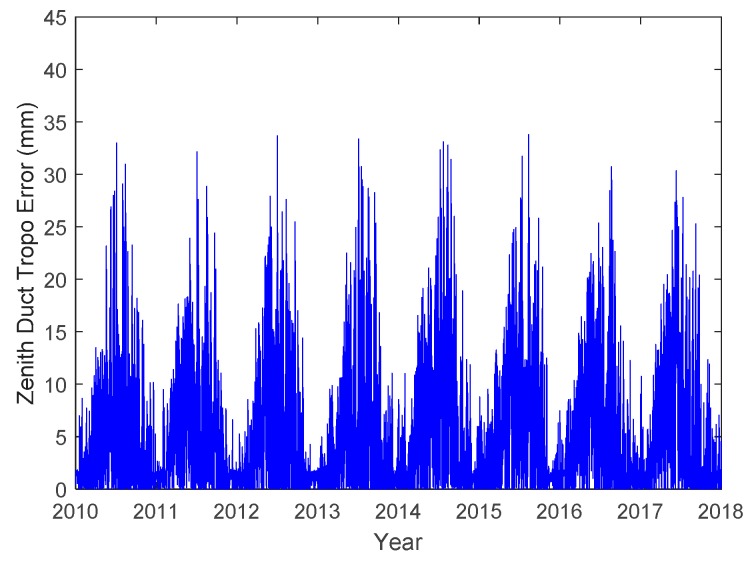
Zenith duct errors at the simulated location (120.3° E, 39.9° N).

The simulation below is conducted at a location (120.3° E, 39.9° N) in the Bohai Sea. Figure 9 shows the zenith tropospheric duct errors at the simulated location from 2010 to 2017. The seasonal variation pattern of the duct errors is prominent and similar every year, and the errors in the summer are generally larger than those in the other three seasons.

### 3.2. Non-Nominal Troposphere

**Figure 10:**
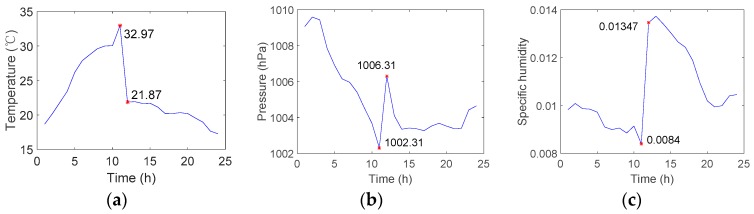
Temperature (**a**); pressure (**b**); and specific humidity (**c**) conditions for location (50.7° N, 75.6° E) on 11 June 2016.

**Figure 11:**
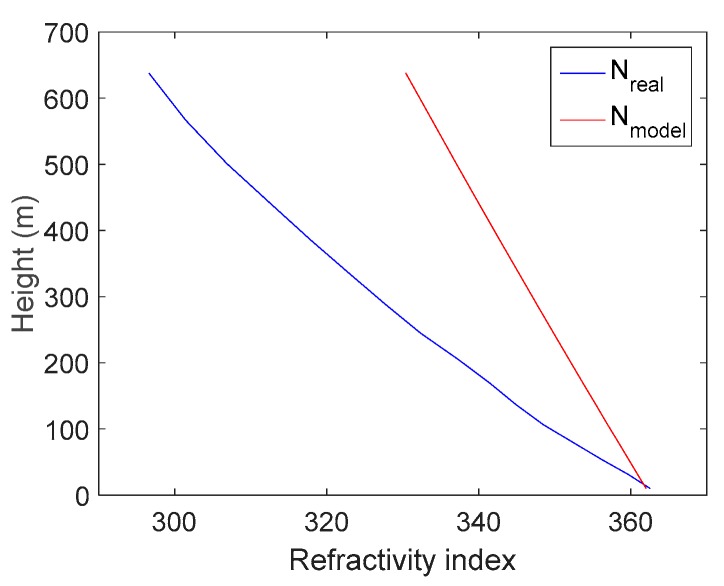
Duct anomaly observed at 12:00 for location (50.7° N, 75.6° E) on 11 June 2016.

An analysis of the ECMWF data indicates that the duct and non-nominal troposphere anomalies can occur simultaneously. Figure 10 shows the temperature, pressure, and specific humidity conditions for location (50.7° N, 75.6° E) on 11 June 2016. The troposphere meteorological parameters fluctuate remarkably from 11:00 to 12:00, which is indicative of the non-nominal troposphere. Figure 11 shows the duct anomaly observed at 12:00 on that day. In addition, Table 1 shows the meteorological conditions and refractivity gradients at a location (120.3° E, 39.9° N) in the Bohai Sea when the change in temperature exceeds 5 °C/h. There are 7 samples of 10 for which the absolute refractivity gradient exceeds 100/km.

The non-nominal tropospheric error calculation process is as follows:

1. Calculate the maximum hourly changes in temperature, pressure, and specific humidity.
2. Use the above values to establish the Weather Wall parameters.
3. Calculate the bounds on the tropospheric delay difference between the ship and aircraft using the Weather Wall model and the Modified Hopfield model [2], which are non-nominal tropospheric errors.

The maximum hourly changes of temperature, pressure, and specific humidity in the Bohai Sea and Yellow Sea from 2010 to 2017 are calculated based on ECMWF data to be 12.77 °C/h, 13.21 hPa/h and 0.0078/h, respectively. Since the input parameter of the modified Hopfield model is the relative humidity, the specific humidity change is converted to a relative humidity change, being approximately 48% [2,15]. 

Based on the above meteorological data analysis, the troposphere weather parameters are as follows:

1. Nominal weather conditions: T_w_ = 33 °C, P_w_ = 990 hPa, RH_w_ = 50%. The temperature lapse rate is set to −6.5 K/km.
2. Weather wall conditions: T_0_ = 20 °C, P_0_ = 1004 hPa, RH_0_ = 100%. The temperature lapse rate is set to −6.5 K/km.

The non-nominal tropospheric errors are related to the satellite elevation and the horizontal distance between the aircraft and the ship station.

The horizontal distance between the aircraft and the ship station is the sum of the distance from the aircraft to the runway threshold and the distance from the landing threshold point to the ship station in the worst case. For GBAS, the landing threshold point is approximately 5 km or 10 km from the GBAS reference station [20]. However, in Sea-Based JPALS, the ship deck area is limited. The lengths of the United States Gerald R. Ford class aircraft carrier, the United States Nimitz class nuclear-powered aircraft carrier, and the Chinese “Liaoning” aircraft carrier are approximately 335 m, 332.85 m, and 304.5 m, respectively [21,22,23], so the distance from the landing threshold point to the ship station is assumed to be 300 m. In addition, the approach of the aircraft from a height of approximately 500 m from the landing point and a horizontal distance of approximately 17.7 km is considered.

**Figure 12:**
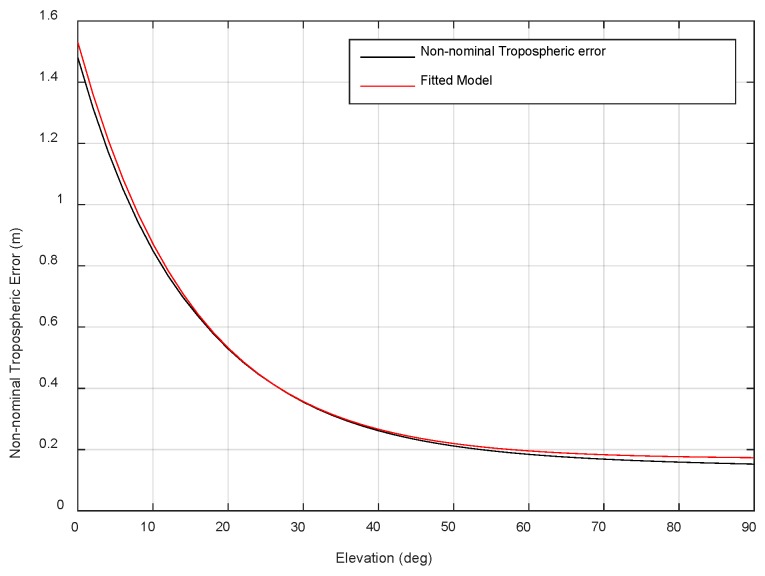
Non-nominal tropospheric error and fitted model.

Therefore, the non-nominal tropospheric error when the horizontal distance between the aircraft and the ship station is approximately 18 km is calculated and then fitted to an exponential function of the satellite elevation *θ*, as shown in Figure 12.

The black line in Figure 12 represents the non-nominal tropospheric errors at different elevation ranges, while the red line represents the fitted model as the following equation.

(6) $$\mu =1.36\times \exp(-\frac{\theta }{15.11})+0.17$$

The three constants in the model can be broadcast by the ship to the aircraft as integrity parameters. Then, the fitted model can be constructed on the aircraft and used to estimate the bound value of the non-nominal error for the satellites affected by the Weather Wall. 

**Figure 13:**
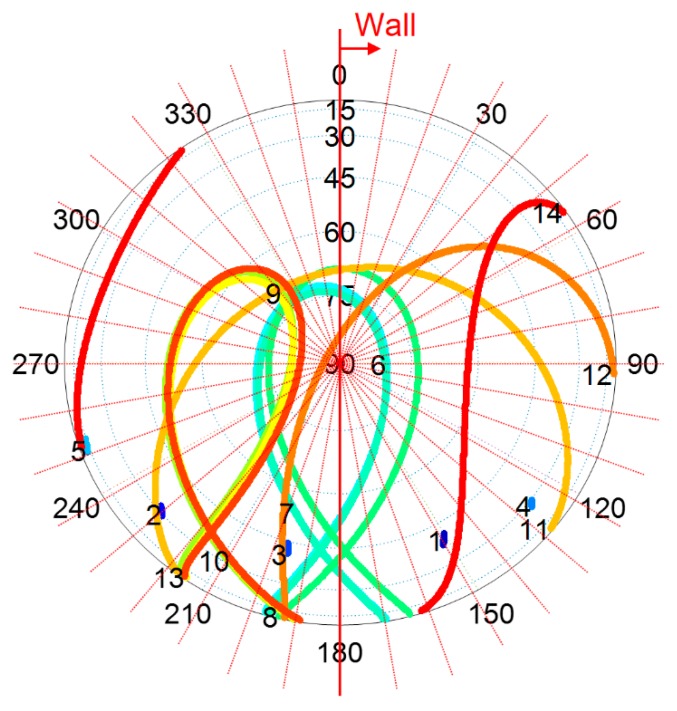
Searching for the azimuth of the Weather Wall in steps of 10°. The skyplot of the simulated location over the simulated day is for BeiDou Navigation Satellite System (BDS).

The satellites whose signals pass through the Weather Wall will be affected by the non-nominal troposphere. To select the affected satellite subset *Q*, the azimuth of the wall needs to be determined. As the Weather Wall exists at one side of the aircraft and the specific azimuth of the wall is not certain, we search for the azimuth of the wall in steps of 10°, as shown in Figure 13, considering the worst satellite geometry possible. The azimuth is selected where the value of $\sum_{Q}S_{\mathrm{vert}}\times \mu$ is maximal in each epoch [10]. *S*_vert_ is the vertical component of the geometry matrix for the visible satellite [24]. *μ* is the bound value of the non-nominal tropospheric error calculated using the fitted model.

## 4. VPL under Tropospheric Anomalies

The errors caused by tropospheric anomalies are non-zero mean errors. The errors will decrease as the distance between the aircraft and the ship decreases. However, when the distance between the aircraft and the ship is not small enough, the errors cannot be eliminated during the differential correction process. Therefore, the error sources should be bounded in integrity monitoring.

The VPL is an important indicator for assessing the Sea-Based JPALS performance. The VPL provides a confidence boundary to bound the positioning error with a large probability (defined by an integrity risk of less than 10^−7^) [9].

The protection level calculation methods under tropospheric anomaly hypothesis proposed previously are based on the least squares method. However, because of the highly stringent requirements for sea-based applications, Sea-Based JPALS is generally based on carrier-phase differential positioning and Kalman filtering [9]. Therefore, a protection level calculation method under the tropospheric anomaly hypothesis based on Kalman filtering is proposed.

In 2004, Frank Van Graas proposed an alternate VPL methodology that bounds the zero mean error and non-zero mean bias error separately [25]. This VPL consists of the component *VPL*_non_ bounding zero mean errors and the component *VPL*_bias_ bounding bias errors.

(7) $$VPL=VPL_{\mathrm{non}}+VPL_{\mathrm{bias}}$$

### 4.1. VPL_non_ Calculation

The nominal errors under the fault-free hypothesis (H0) are assumed to follow a zero-mean Gaussian distribution and are bounded by the component *VPL*_non_. The ship subsystem receiver failures are protected by additional protection levels, which are not considered in this paper.

For Sea-Based JPALS, Samer Khanafseh and Boris Pervan proposed two types of positioning methods based on a Kalman filter: the averaging approach and the coupled estimation approach [13]. The coupled estimation approach was found to provide better performance than the averaging method. Therefore, the *VPL*_H0_ calculation method for the coupled estimation approach is used to calculate *VPL*_non_.

For the fault-free missed detection multiplier *K*_ffmd_, the method in Ref. [13] does not consider the incorrect fix risk of the integer ambiguity in carrier-phase measurements. Therefore, the multiplier *K*_ffmd_ is recalculated considering the probability of incorrect fix (*P*_IF_) of the integer ambiguity [26].

When the allocated integrity risk is 10^−7^, *VPL*_non_ is defined by

(8) $$\mathrm{Prob}\left\{\left|{\hat{x}}_{\mathrm{vert}}-x_{\mathrm{vert}}\right|>VPL_{\mathrm{non}}|CF\right\}\cdot \left(1-P_{\mathrm{IF}}\right)+\mathrm{Prob}\left\{\left|{\hat{x}}_{\mathrm{vert}}-x_{\mathrm{vert}}\right|>VPL_{\mathrm{non}}|IF\right\}\cdot P_{\mathrm{IF}}=10^{-7}$$

where $x_{\mathrm{vert}}$ is the true value of the vertical positioning component ${\hat{x}}_{\mathrm{vert}}$, CF indicates that the integer ambiguity is fixed correctly, and IF indicates an incorrect fix.

Given an incorrect fix, it is assumed that the resulting position error will generally be large. Therefore, we obtain

(9) $$\mathrm{Prob}\left\{\left|{\hat{x}}_{\mathrm{vert}}-x_{\mathrm{vert}}\right|>VPL_{\mathrm{non}}|IF\right\}\approx 1$$

(10) $$\mathrm{Prob}\left\{\left|{\hat{x}}_{\mathrm{vert}}-x_{\mathrm{vert}}\right|>VPL_{\mathrm{non}}|CF\right\}=\frac{10^{-7}-P_{\mathrm{IF}}}{1-P_{\mathrm{IF}}}$$

so that *VPL*_H0_ is

(11) $$VPL_{\mathrm{non}}=K_{\mathrm{ffmd}}\left(P_{\mathrm{IF}}\right)\cdot \sigma_{\mathrm{vert}|\mathrm{CF}}$$

where $\sigma_{\mathrm{vert}|\mathrm{CF}}$ is the vertical position error standard deviation obtained from the variance-covariance matrix of the relative positioning solution based on the Kalman filter. If *P*_IF_ is assumed to be less than 10^−8^, then *K*_ffmd_ is approximately 5.35.

### 4.2. VPL_bias_ Calculation

The non-zero mean vertical positioning bias errors caused by tropospheric anomalies are bounded by *VPL*_bias_.

For the duct anomaly, the worst-case zenith duct error is used as a bound value *γ* for all zenith duct errors *d*.

(12) |d|≤γ

Multiplying the zenith error *γ* by the vector **a** of the obliquity factors for visible satellites results in the duct error vector $\mu_{\mathrm{duct}}$ in the measurement domain.

(13) $$\mu_{\mathrm{duct}}=a\gamma$$

For the non-nominal troposphere, the non-nominal tropospheric errors are calculated using the fitted exponential model in the section above. Then, the vector $\mu_{\mathrm{non}-\mathrm{nominal}}$ of non-nominal tropospheric errors for visible satellites is obtained. Note that the errors of the satellites not impacted by the Weather Wall are zero.

If the duct and non-nominal troposphere anomalies are simultaneously taken into account, then the total tropospheric error vector $\mu_{\mathrm{tropo}}$ is

(14) $$\mu_{\mathrm{tropo}}=\mu_{\mathrm{non}-\mathrm{nominal}}+\mu_{\mathrm{duct}}$$

In Sea-Based JPALS, the relative positions of the ship and the aircraft are calculated based on the Kalman filter. For the tropospheric delay on path 1, the TC model is used to correct the ship measurements. Then, the measurements of the ship and the aircraft are double differenced [17]. Since **μ**_tropo_ is the residual tropospheric error in single differenced measurements between the ship and aircraft, the residual tropospheric error $\Delta \mu_{\mathrm{tropo}}$ in double-differenced measurements is the difference between the residual tropospheric errors $\mu_{\mathrm{tropo}}$ of different satellites.

(15) $$\Delta \mu_{\mathrm{tropo}}=\left[\begin{array}{c} \mu_{\mathrm{tropo}}^{1}-\mu_{\mathrm{tropo}}^{m}\\ \begin{array}{c} \vdots \\ \mu_{\mathrm{tropo}}^{m-1}-\mu_{\mathrm{tropo}}^{m}\\ \begin{array}{c} \mu_{\mathrm{tropo}}^{m+1}-\mu_{\mathrm{tropo}}^{m}\\ \vdots \\ \mu_{\mathrm{tropo}}^{n}-\mu_{\mathrm{tropo}}^{m} \end{array} \end{array} \end{array}\right]$$

where *n* is the number of available satellites. *m* represents the reference satellite, which generally has the highest elevation.

Based on the standard equation for updating the state vector $\delta \hat{x}$ in a Kalman filter, the positioning bias error caused by the tropospheric anomalies is estimated as follows:

(16) $$\delta \hat{x}\left(k|k\right)=\delta \hat{x}\left(k|k-1\right)+K\left(k\right)\left(\Delta \mu_{\mathrm{tropo}}\left(k\right)-H\left(k\right)\cdot \delta \hat{x}\left(k|k-1\right)\right)$$

where K(k) is the Kalman filter gain matrix of the *k*-th state and H(k) is the measurement matrix of the *k*-th state.

By extracting the element that corresponds to the vertical component $\delta {\hat{x}}_{v}$ from $\delta \hat{x}$, *VPL*_bias_ under the duct hypothesis is computed as

(17) $$VPL_{\mathrm{bias}}=\delta {\hat{x}}_{v}$$

Then, the VPL under the duct hypothesis is computed as

(18) $$VPL=\delta {\hat{x}}_{v}+K_{\mathrm{ffmd}}\left(P_{\mathrm{IF}}\right)\cdot \sigma_{\mathrm{vert}|\mathrm{CF}}$$

## 5. Simulations

### 5.1. Simulation Options and Parameters

**Figure 14:**
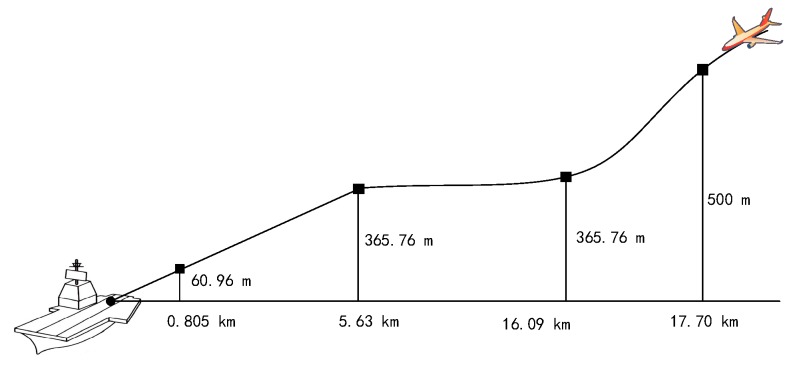
Simulation area of aircraft landing.

In this work, a straight-in approach (Case-III landing approach) is assumed [26,27]. The simulation area begins with the aircraft being 500 m above the ship and the horizontal distance being approximately 17.70 km, as shown in Figure 14.

A given approach is said to be available if the integrity requirements are satisfied at each point along the approach. In this paper, the vertical alert limit (VAL) for Sea-Based JPALS is set to be 1.8 m according to reference [11].

To account for the GNSS satellite geometry change, the simulation duration is one day, and the interval is 10 s. 

For the duct anomaly, the zenith error is set to be 45.64 mm at a 500 m height and decreases linearly with the height during the approach. For the non-nominal troposphere, the error decreases linearly with the horizontal distance between the aircraft and the ship.

The parameters of the TC uncertainty are obtained by calculating the annual mean value and standard deviation of the data at the simulated location in 2017. The tropospheric scale height is 7583.9 m, the refractivity index is 328.6, and the refractivity index uncertainty is 29.5.

Simulation options:

1. Location: 120.3° E, 39.9° N.
2. Integrity risk: 10^−7^ [9].
3. Constellations: current GPS constellation and current BDS constellation.
4. Multipath and receiver noise error model: both code and carrier errors were modeled as first-order Gauss-Markov measurement error models. The time constants in the model for the ship and aircraft are 60 s and 30 s, respectively [11,28].
5. Standard deviation of pseudorange measurement error: 0.35 m.
6. Standard deviation of carrier-phase measurement error: 0.007 m.
7. Mask angle: 7.5° (accounting for potential obstructions in the shipboard environment and loss of low-elevation lines of sight at the aircraft due to attitude motion [26]).

### 5.2. Results Analysis

**Figure 15:**
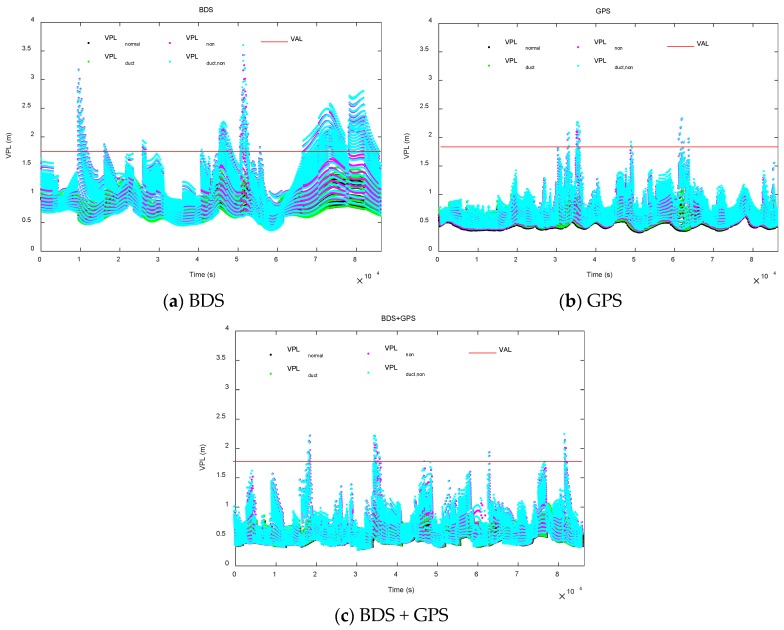
Vertical Protection Level (VPL) simulation results under BDS/Global Positioning System (GPS)/BDS + GPS constellations.

Figure 15 shows the VPL simulation results based on the above options. 

*VPL*_normal_ is the VPL under the fault-free hypothesis. 

*VPL*_duct_ is the VPL when only the duct anomaly is considered.

*VPL*_non_ is the VPL when only the non-nominal troposphere is considered.

*VPL*_duct,non_ is the VPL when both the duct anomaly and the non-nominal troposphere are considered.

According to Table 2, the average VPL increment caused by only the duct anomaly is 0.082 m with a growth percentage of 12.903%; the average VPL increment caused by only the non-nominal troposphere is 0.211 m with a growth percentage of 30.857%. The average VPL increment caused by the two tropospheric anomalies is 0.272 m with a growth percentage of 40.427%.

Figure 15 and Table 2 indicate that the following inequality can be obtained:

(19) $$VPL_{\mathrm{duct},\mathrm{non}}>VPL_{\mathrm{non}}>VPL_{\mathrm{duct}}>VPL_{\mathrm{normal}}$$

The inequality indicates that the introduction of non-nominal tropospheric and duct anomaly errors increases VPL and that the impact of non-nominal tropospheric errors is greater than that of duct anomaly errors.

For availability, if the protection level exceeds the alarm limit at some point during the approach, the approach is considered unavailable. The typical availability requirement for Sea-Based JPALS specified in references [9,26] is 99.7%. To calculate the average availability, the simulation time is extended to 10 days. The results are shown in Table 3. Under the fault-free hypothesis, the average availability is 100%. When the duct anomaly and non-nominal troposphere are considered separately, the average availabilities are 99.983% and 96.326%, respectively. Although the loss of availability is small when only the duct anomaly is considered, when the two tropospheric anomalies are considered simultaneously, the average availability is 95.257%, with a loss of 4.743% relative to fault-free availability.

From Table 2 and Table 3, it is found that the impact of tropospheric anomalies under the BDS constellation is greater than that under the GPS constellation. There may be two main reasons. First, for the simulated location, the elevations of the five BDS GEO satellites are all below 45°, and thus the duct errors of the GEO satellites are relatively large. Second, during the simulation, the percent of the epochs when the five BDS GEO satellites (PRN 1 to PRN 5) are affected by the non-nominal troposphere are 98.83%, 98.69%, 100%, 90.39%, and 85.20%, respectively. The likelihood that the five satellites are all affected is 75.59%. And the non-nominal tropospheric errors of these GEO satellites are large due to the low elevations.

## 6. Conclusions

An analysis based on the published 8-year historical meteorological data of China and the surrounding areas from ECMWF shows that the duct anomaly and non-nominal troposphere can occur simultaneously. 

For the duct anomaly, the maximum zenith error is observed to be 45.64 mm, and the duct error has a prominent seasonal characteristic in that the errors in the summer are generally larger than those in the other three seasons. For the non-nominal troposphere, the worst meteorological conditions in China’s surrounding sea areas are used to calculate non-nominal errors, and then a satellite-elevation-dependent fitting model is obtained. Finally, a protection level calculation method based on Kalman filtering under tropospheric anomalies is proposed, and the simulation of multiple landing approaches is conducted.

The results show that the average VPL increments introduced by the duct and non-nominal troposphere anomalies are 0.082 m and 0.211 m, respectively; the growth percentages are 12.903% and 30.857%, respectively. The average VPL increments under simultaneous anomalies is 0.272 m with a growth of 40.427%. Tropospheric anomaly hypotheses cause some loss of system availability. The average availability of the system under normal conditions is 100%. For the duct anomaly and the non-nominal troposphere the availability loss is 0.017% and 3.674%, respectively. Under simultaneous anomalies, the availability loss is 4.743%. The simultaneous occurrence of tropospheric anomalies is expected to adversely affect the Sea-Based JPALS integrity and availability.

The above results are somewhat conservative, mainly in regard to two aspects. First, the maximum zenith duct error obtained based on the 8-year meteorological data of China and the surrounding areas is used in the simulation. In practical applications, different error bound values can be set according to the specific season and location. Second, the azimuth of the Weather Wall in the simulation is selected considering the worst satellite geometry possible. The detection method of this azimuth needs further study.

The protection level calculation method proposed in this paper can bound the errors caused by tropospheric anomalies, but this method requires the modification of the airborne integrity architecture. In future work, we plan to develop a method accounting for tropospheric anomalies without modifying the airborne architecture in Sea-Based JPALS.

## Figures and Tables

**Table 1:** Meteorological conditions and refractivity gradients at location (120.3° E, 39.9° N).

Time	Temperature Change (°C/h)	Pressure Change (hPa/h)	Specific Humidity Change (/h)	Absolute Refractivity Gradient (/km)
31 July 2010 05:00	6.4928	1.461	0.0050	117.56
14 April 2011 19:00	5.8820	1.4481	0.0016	89.124
17 March 2013 20:00	11.057	3.68	0.0027	63.027
4 August 2013 17:00	5.0482	1.9732	0.0029	103.06
23 September 2013 03:00	5.2567	1.3430	0.0036	114.7
25 October 2014 18:00	5.0274	1.8183	0.0023	123.31
21 March 2015 17:00	6.9922	2.6325	0.0013	186.97
15 April 2015 14:00	5.6072	1.5774	0.00095	120.00
16 February 2017 00:00	5.5364	1.9954	0.0013	86.49
11 March 2017 15:00	5.0696	1.4906	0.000637	140.6
31 July 2010 05:00	6.4928	1.461	0.0050	117.56
14 April 2011 19:00	5.8820	1.4481	0.0016	89.124

**Table 2:** Average VPL increments and growth percentages under tropospheric anomalies.

VPL Increments (m)/Percentage	BDS	GPS	BDS + GPS
Duct anomaly	0.085/9.98%	0.083/14.23%	0.079/14.50%
Non-nominal troposphere	0.332/38.90%	0.127/21.77%	0.173/31.90%
Both anomalies	0.398/46.57%	0.188/32.39%	0.230/42.32%

**Table 3:** Average availability results under tropospheric anomalies for 10 days (86,400 epochs).

Number of Unavailable Epochs/Average Availability	BDS	GPS	BDS + GPS
Nominal	0/100%	0/100%	0/100%
Duct anomaly	43/99.950%	0/100%	0/100%
Non-nominal troposphere	8700/89.931%	397/99.541%	427/99.506%
Both anomalies	11250/86.979%	476/99.449%	567/99.344%
